# *Cerebrospinal Fluid Research*: The first six months and the introduction of article processing charges

**DOI:** 10.1186/1743-8454-2-4

**Published:** 2005-07-19

**Authors:** Hazel C Jones

**Affiliations:** 1 Editorial Office Gagle Brook House, Chesterton, Bicester, Oxon OX26 1UF, United Kingdom

## Abstract

Article processing charges (APCs) have recently been introduced for authors submitting papers to *Cerebrospinal Fluid Research*. This editorial is to inform readers about the need and use of APCs and about the advantages of free open access publishing.

## Introduction

It is now over six months since the launch of *Cerebrospinal Fluid Research *in December 2004. *Cerebrospinal Fluid Research *is published by BioMed Central, an independent publisher committed to ensuring peer-reviewed biomedical research is Open Access. To fund this, from July 1^st ^2005 authors of articles accepted for publication will be asked to pay an article-processing charge (APC) of £330.00.

Traditionally, readers pay to access articles, either through subscriptions or by paying a fee each time they download an article. Escalating journal subscriptions and shrinking library budgets have resulted in libraries subscribing to fewer journals [1], and the range of articles available to readers is becoming more limited. Although traditional journals publish authors' work for free (unless there are page or colour charges), paying to access articles limits the number of people that can read, use and cite them. The benefits of Open Access and of *Cerebrospinal Fluid Research*'s Open Access policy were highlighted in a previous editorial [2].

## Description of Payment

APCs will allow continued Open Access to all of *Cerebrospinal Fluid Research*'s articles. Authors are asked to pay £330 if their article is accepted for publication. Waiver requests will be considered on a case-by-case basis, by the Editor-in-Chief. Authors can circumvent the charge if their institution becomes a 'member' of BioMed Central, in which case the annual membership fee covers the APCs for the authors of all BioMed Central journals at that institution for that year. Current members include NHS England, the World Health Organization, the US National Institutes of Health, Harvard, Princeton and Yale universities, and all UK universities [3]. No charge is made for articles that are rejected after peer review. Many funding agencies have also realized the importance of Open Access publishing and have specified that their grants may be used directly to pay APCs [4]. Additionally, the UK Research Councils recently announced that they are considering their position on improved access to research outputs [5].

## What is your APC used for?

The APC pays for online submission and efficient peer review, for the article to be freely and universally accessible in various formats online, and for the processes required for inclusion in PubMed and archiving in PubMed Central, e-Depot, Potsdam and INIST. There is no remuneration of any kind provided to the Editor-in-Chief, to any members of the editorial board, or to peer reviewers; all of whose work is entirely voluntary. Although some authors may consider £330 expensive, it must be remembered that *Cerebrospinal Fluid Research *does not levy additional page or colour charges on top of this fee. With the article being online only, any number of colour figures and photographs can be included, at no extra cost. Another common expense with traditional journals is the purchase of reprints for distribution, and the cost of these reprints is frequently greater than our APCs. *Cerebrospinal Fluid Research *provides free, publication-quality pdf files for distribution, in lieu of reprints.

## Advantages of Open Access

Although several journals now offer free online access to their articles, this is different from Open Access (as defined by the Bethesda Statement [6]). Journals often delay free access for 6–12 months, and even when the full text is available, readers are not allowed to reproduce and/or disseminate the work because of restrictions imposed by the copyright policy. That said, *Cerebrospinal Fluid Research *is not alone in the move to Open Access funded by APCs: many publishers now offer various "hybrid" forms where the authors are asked to contribute to the publishing costs. The Public Library of Science also has Open Access journals that are funded by grants and APCs of about US$1500 per article [7]. The high profile of these journals will raise awareness of Open Access and encourage researchers in all disciplines to understand and accept Open Access, with APCs as an acceptable method to fund it.

## Conclusion

By providing a forum for Open Access, APCs will enable *Cerebrospinal Fluid Research *to continue to publish attractive and important papers on outstanding research that will be accessible to everyone worldwide. We believe this process will bring together people in a vigorous field of research, and we hope you will support this progress by submitting your next article to this Open Access journal.

## References

[B1] Tamber PS (2000). Is scholarly publishing becoming a monopoly?. BMC News and Views.

[B2] Jones HC (2004). *Cerebrospinal Fluid Research: *A new platform for dissemination of research, opinions and reviews with a common theme,. Cerebrospinal Fluid Research.

[B3] BioMed Central Institutional members. http://www.biomedcentral.com/inst/.

[B4] Which funding agencies explicitly allow direct use of their grants to cover article processing charges?. http://www.human-resources-health.com/info/faq/apcfaq.asp?txt_faq=grants.

[B5] Research Councils UK: Access to Research Outputs. http://www.rcuk.ac.uk/access/index.asp.

[B6] Bethesda Statement on Open Access Publishing. http://www.earlham.edu/~peters/fos/bethesda.htm.

[B7] Public Library of Science. http://www.plos.org/faq.html#pubfees.

